# Japan’s Growing Public Health Crisis: Tackling the Alarming Increase in Streptococcal Toxic Shock Syndrome

**DOI:** 10.7759/cureus.75890

**Published:** 2024-12-17

**Authors:** Harendra Kumar, Arkadeep Dhali, Gopal Krishna Dhali

**Affiliations:** 1 General Medicine, Dow University of Health Sciences, Karachi, PAK; 2 Internal Medicine, Sheffield Teaching Hospitals National Health Service (NHS) Foundation Trust, Sheffield, GBR; 3 Gastroenterology, School of Digestive and Liver Diseases, Institute of Postgraduate Medical Education and Research, Kolkata, IND

**Keywords:** group a streptococcus pyogenes, japan stss outbreak 2024, outcome, public health and safety, streptococcal toxic shock syndrome

## Abstract

Japan is experiencing a dramatic spike in streptococcal toxic shock syndrome (STSS) cases, exceeding the previous year’s statistics. This life-threatening illness, caused by *Streptococcus pyogenes*, has been connected not only to the relaxation of COVID-19 precautions but also to the prolonged effects of confinement and lack of contact with the surrounding environment/ecology. The condition is characterized by a sudden onset of symptoms, including high fever, rash, and shock, and demands immediate medical intervention. This alarming increase emphasizes the need for heightened public awareness and prompt medical responses. Strengthening surveillance, improving diagnostic capabilities, and ensuring swift treatment are critical steps to manage this escalating health crisis and prevent future cases effectively.

## Editorial

**Figure 1:**
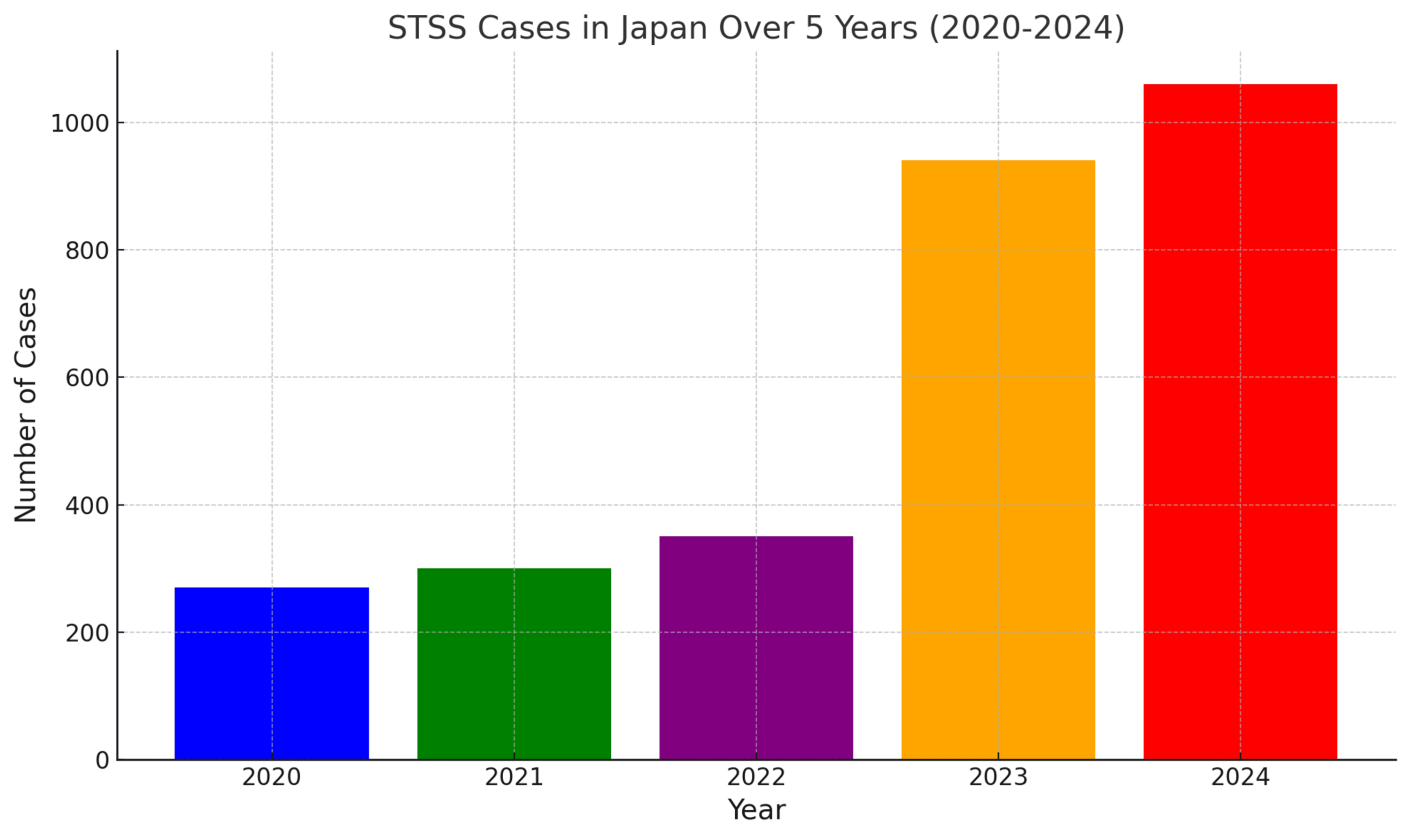
Streptococcal toxic shock syndrome (STSS) cases in Japan over five years (2020-2024).

The sharp rise in streptococcal toxic shock syndrome (STSS) cases in Japan has become a pressing public health concern. With over 1,060 cases reported since January 2024, surpassing the 941 cases reported for the entire previous year, this represents a 12.6% increase (Figure 1) [1,2]. This surge requires immediate and targeted intervention from health authorities and the public. However, critical details about the affected population remain underreported. It is essential to determine whether the surge predominantly affects older adults with weakened immunity, school-aged children exposed through contact with potential reservoirs, or young women, who may face specific exposure risks. Understanding these demographic patterns is crucial for developing effective prevention strategies, prioritizing healthcare resources, and curbing the impact of this life-threatening condition [2,3].

*Streptococcus pyogenes*, a gram-positive bacteria, causes STSS [1]. It is a severe and sometimes fatal sickness caused by the release of exotoxins, such as streptococcal pyrogenic exotoxins (SPEs), which act as superantigens and trigger a massive systemic inflammatory response. This response can cause widespread tissue damage, shock, and multiple organ failure [1,2]. The disease usually follows a localized infection, such as cellulitis, pharyngitis, or wound infections, but it can also occur in otherwise healthy people.

The cause of STSS is immune system overactivation, which results in the release of pro-inflammatory cytokines that cause vascular leakage, hypotension, and multi-organ failure [2]. The body's response to these poisons results in endothelial damage, poor tissue perfusion, and an impaired immune response, all of which contribute to the illness's rapid progression [3,4].

Clinically, STSS manifests as a sudden onset of fever, chills, muscle aches, nausea, vomiting, and tiredness [1-3]. As the sickness progresses, people may experience hypotension, confusion, and erythematous rashes that look like sunburn [4]. In severe cases, necrotizing fasciitis may develop, and the patient's health may often deteriorate rapidly, leading to shock and organ failure [2]. Renal failure, respiratory distress, and disseminated intravascular coagulation (DIC) are common complications of STSS [3]. The disease's high mortality rate, which can approach 30%, underscores the importance of early discovery and treatment. Intravenous antibiotics, such as penicillin or clindamycin, are frequently required for effective therapy, as is supportive care, such as fluid resuscitation and organ support [3-5].

Given STSS's rapid progression and high mortality rate, early detection and treatment are critical in managing the disease and improving patient outcomes [1,3]. The growing number of cases necessitates increased public health efforts to ensure timely response and prevent future illness transmission [2].

The exact cause of the sharp increase in STSS cases remains unclear. Some researchers suggest the surge may be linked to the loosening of COVID-19 restrictions [1-3]. During the pandemic, strict hygiene standards and social distancing measures were in place, effectively reducing the spread of various infectious diseases, including STSS [2,4]. For example, a study comparing the incidence of invasive Group A streptococcal (iGAS) infections, which include STSS, found that the case rate before the COVID-19 pandemic was significantly lower. Specifically, the case rate was 205 per 100,000 ICU admissions before the pandemic, compared to 949 per 100,000 ICU admissions after the pandemic [2,5,6]. Additionally, another study observed that during the pre-COVID-19 period (January 2015-March 2020), the number of invasive Group A Streptococcal (iGAS) cases was much lower compared to the outbreak period (October 2022-April 2023) [2,3]. Ongoing investigations are looking into changes in bacterial strains, environmental factors, and human behavior as potential contributors to the outbreak. Regardless of the cause, swift action by health authorities is essential to address this serious public health concern [4].

Public awareness campaigns are crucial for educating the public about the symptoms and risks associated with STSS. Encourage individuals exhibiting symptoms like fever, chills, nausea, vomiting, diarrhea, and rashes to seek immediate medical attention [1]. Healthcare providers must also be vigilant in diagnosing and treating STSS early to prevent severe outcomes [2,5].

Enhanced surveillance is vital for controlling the outbreak. Health authorities should implement robust monitoring systems to track STSS incidence and identify potential clusters [6,7]. This data will assist in developing targeted interventions and understanding the disease's epidemiology. Further research into bacterial virulence, host vulnerability, and environmental factors is also necessary to uncover the root causes of this surge [8].

For individuals affected by STSS, early medical intervention is key. The prompt administration of antibiotics and supportive care can significantly improve patient outcomes [1,5]. Consequently, healthcare facilities should be equipped to manage the increasing number of cases, and medical professionals should be trained to treat STSS effectively. The government must ensure that healthcare institutions are equipped with the necessary resources to manage STSS cases effectively, including adequate supplies of intravenous antibiotics, critical care beds, and trained personnel to handle severe complications [8]. Additionally, rapid diagnostic tools and public health outreach programs should be prioritized to facilitate early detection and prevent further transmission [6,7].

In addition to these measures, public awareness campaigns should emphasize behaviors and practices that minimize the risk of infection. Regular hand washing, proper wound care, and avoiding close contact with infected individuals are simple but effective ways to prevent the spread of the bacteria [3]. Schools, workplaces, and community centers can play a significant role in disseminating this information and promoting best practices [2].

The surge in STSS cases represents a public health emergency that requires a coordinated response from all stakeholders. Collaboration is essential to mitigate the outbreak's impact and safeguard the well-being of communities. The government, healthcare institutions, and the public must collaborate to implement effective strategies to control the spread of STSS and prevent further cases.

The Japanese Ministry of Health, Labor, and Welfare is actively raising awareness about STSS, advising the public to recognize symptoms such as fever, chills, nausea, vomiting, diarrhea, and rashes and to seek prompt medical attention [1-3]. Enhanced surveillance systems are being developed to monitor case numbers, track STSS incidence, and identify potential clusters [2,3]. Prophylactic measures (Table 1) are being explored for high-risk individuals, especially those in close contact with confirmed STSS cases [3,8]. Public health messages continue to reinforce good hygiene practices, such as frequent hand washing, proper wound care, and avoiding direct contact with sick individuals, through schools, businesses, and community settings [3,4].

**Table 1:** Recommendations for addressing the streptococcal toxic shock syndrome (STSS) outbreak in Japan.

Recommendation	Description	Key actions
Public awareness campaigns	Educate the public about STSS symptoms and the importance of seeking early care.	Launch media campaigns. Promote awareness in schools, workplaces, and communities.
Enhanced surveillance	Implement stronger monitoring systems to track and respond to STSS cases.	Set up case tracking and reporting systems. Identify clusters of infections.
Research into outbreak causes	Investigate the factors contributing to the surge in STSS cases.	Study bacterial strain changes. Analyze environmental and behavioral factors.
Prompt medical treatment	Ensure quick and effective medical care to improve patient outcomes.	Equip healthcare facilities for STSS treatment. Train healthcare professionals.
Government resource allocation	Allocate resources to manage the growing number of STSS cases.	Increase funding for healthcare resources. Provide necessary medical supplies.
Hygiene promotion and prophylactic measures	Promote hygiene practices to prevent STSS and consider preventive actions for high-risk individuals.	Encourage hand washing, wound care. Consider prophylactic antibiotics for high-risk contacts.
Stakeholder collaboration	Coordinate efforts between government, healthcare organizations, and the public.	Develop a national response plan. Facilitate communication across sectors.

Conclusion

The rapid growth of STSS patients in Japan should raise serious concerns. Solving this public health problem demands a timely reaction. We can battle this epidemic and protect our society's health by raising public awareness, improving monitoring, providing prompt medical care, and taking preventative measures. We encourage the government to make this a high priority and act immediately to safeguard our communities from the deadly impacts of STSS. It is our collective obligation to act promptly and forcefully to stop the spread of STSS and mitigate its repercussions in our nation.
